# PDGF-AA Promotes Osteogenic Differentiation and Migration of Mesenchymal Stem Cell by Down-Regulating PDGFRα and Derepressing BMP-Smad1/5/8 Signaling

**DOI:** 10.1371/journal.pone.0113785

**Published:** 2014-12-03

**Authors:** Anna Li, Xuechun Xia, James Yeh, Huiyi Kua, Huijuan Liu, Yuji Mishina, Aijun Hao, Baojie Li

**Affiliations:** 1 Department of Histology and Embryology, Shandong University School of Medicine, 44 Wenhua Xi Road, Jinan, Shandong, 250012 P.R. China; 2 The Bio-X Institutes, Key Laboratory for the Genetics of Developmental and Neuropsychiatric Disorders, Ministry of Education, Shanghai Jiao Tong University, Shanghai 200030, China; 3 The Institute of Molecular and Cell Biology, Agency for Science, Technology, and Research, Singapore 138632, Singapore; 4 Department of Biologic and Materials Sciences, School of Dentistry, University of Michigan, Ann Arbor, Michigan, United States of America; Baylor College of Medicine, United States of America

## Abstract

Platelet-derived growth factors (PDGFs) play important roles in skeletal development and bone fracture healing, yet how PDGFs execute their functions remains incompletely understood. Here we show that PDGF-AA, but not -AB or -BB, could activate the BMP-Smad1/5/8 pathway in mesenchymal stem cells (MSCs), which requires BMPRIA as well as PDGFRα. PDGF-AA promotes MSC osteogenic differentiation through the BMP-Smad1/5/8-Runx2/Osx axis and MSC migration via the BMP-Smad1/5/8-Twist1/Atf4 axis. Mechanistic studies show that PDGF-AA activates BMP-Smad1/5/8 signaling by feedback down-regulating PDGFRα, which frees BMPRI and allows for BMPRI-BMPRII complex formation to activate smad1/5/8, using BMP molecules in the microenvironment. This study unravels a physical and functional interaction between PDGFRα and BMPRI, which plays an important role in MSC differentiation and migration, and establishes a link between PDGF-AA and BMPs pathways, two essential regulators of embryonic development and tissue homeostasis.

## Introduction

PDGFs are growth factors that promote cell proliferation and migration. In addition, PDGFs have been shown to regulate cell differentiation, although the underlying mechanisms remain largely unknown [1]–[3]. There are four PDGFs (A–D) that are expressed in tissue-specific manners [1]. PDGF molecules bind to specific cell surface receptor PDGFRs, which are members of receptor tyrosine kinases, to execute their functions [1], [2]. PDGF-AA mainly activates PDGFRα while PDGF-BB activates PDGFRβ. The main signaling pathways downstream of PDGFRs include MAPKs, PI-3K, Stat3, and the Rho/Rac cascades, which control cell proliferation, migration, and survival [3]. PDGFs-elicited signaling events are tightly regulated. One regulatory mechanism is endocytosis and lysosome-mediated degradation of PDGFRs, which requires ligand-binding and PDGFR autophosphorylation. This acts as a feedback regulation mechanism to attenuate PDGF signaling [4], [5]. While endocytosed PDGFRα molecules are quickly degraded, there is evidence to support that endocytosed PDGFβ molecules are recycled back to the cell surface [6].

PDGF-AA is mainly synthesized and secreted by epithelial cells and acts on mesoderm-derived cells, which express PDGFRα. Its main function is to promote mesenchyme expansion in addition to angiogenesis [7]–[9]. PDGFs have an important function in bone development. PDGFRα ablation led to defects in skeletal patterning and maturation [10], [11]. In adults, PDGFs play important roles in wound healing and bone fracture healing, where it act on fibroblasts, MSCs, and other cell types, and as such PDGFs might present a class of therapeutic regents for wound healing and bone regeneration [7], [12], [13]. At the cell level, PDGFs are shown to promote MSC proliferation [14]–[16]. While PDGF-BB was reported to inhibit osteoblast differentiation [3], [17], PDGF-AA's function in osteoblast differentiation is inconclusive [18]–[20]. It remains unclear how PDGFs decide the fate of differentiation or proliferation in MSC.

In this report, we show that PDGF-AA, but not BB, could activate BMP-Smad1/5/8 signaling and thereby promotes MSC osteogenic differentiation via BMP-Smad1/5/8-Runx2/Osx and MSC migration via BMP-Smad1/5/8-Twist1/Atf4. BMPs are growth factors/cytokines that are critical for skeletal development and remodeling and are also used to treat bone fracture ununions [21]. As members of the TGFβ superfamily, BMPs bind to BMPRI and II and activate the Smad1/5/8 pathway to stimulate osteogenic differentiation [22], [23]. We found that PDGF-AA-induced Smad1/5/8 activation goes through BMPRIA and PDGFRα. PDGF-AA induces down-regulation of PDGFRα and knockdown of PDGFRα by interference RNA led to increased Smad1/5/8 activation, which could not be further activated by PDGF-AA. Furthermore, inhibition of lysosome-mediated protein turnover diminished PDGF-AA-induced Smad1/5/8 activation. These results indicate that PDGFRα is a negative regulator of BMP-Smad1/5/8 signaling and that PDGF-AA activates Smad1/5/8 by down-regulating PDGFRα. Mechanistically, PDGFRα interacts with BMPRIA and this interferes with the interaction between BMPRIA and BMPRII, which is necessary for Smad1/5/8 activation [22]. These findings provide a mechanism by which PDGF-AA regulates MSC differentiation and migration and reveal an unidentified connection between PDGF-PDGFRα and BMP-Smad1/5/8 signaling pathways.

## Materials and Methods

### Ethics Statement

Animal experimentation in this study, including normal C57BL/6 mice and Bmpr1a^fl/fl^mice, was carried out in accordance with recommendations in the National Research Council Guide for Care and Use of Laboratory Animals, with the protocols approved by the Institutional Animal Care and Use Committee of Shanghai, China [SYXK (SH) 2011–0112]. All efforts were made to minimize suffering of mice.

### Mice, MSC isolation and culture

Bmpr1a^fl/fl^ mice (on C57BL/6 genetic background) were obtained from Yuji Mishina's Laboratory [24]. Bone marrow derived MSCs were isolated from 8-week old C57BL/6 mice. The bone marrow was collected from femurs and tibiae by flushing with α-MEM culture medium with a 25-gauge needle. Cell suspensions were mixed with a pipette followed by filtering through a 70-mm strainer to remove any large cell clumps or bone particles. The cells were counted using trypan blue staining and cultured in α-MEM medium containing 10% fetal bovine serum, 1% L-glutamine and 1% penicillin-streptomycin (Gibco, Invitrogen Corporation, USA) for expansion. After 3 days, the non-adherent cells were removed from the culture by changing the medium, while the adherent cells were sub-cultured. Medium were replaced every 3 days. After 7 day, cells were harvested for a second *in vitro* expansion. MSCs in passages 2–3 were collected and used for further study.

### Osteogenic differentiation

The MSCs were cultured in the osteogenesis differentiation medium: α-MEM containing 0.5% FBS, 100 µg/ml ascorbic acid and 10 mm β-glycerophosphate (Sigma-Aldrich Corp., St. Louis, MO, USA). Medium was changed every 3 days [25]. After 7 and 14 days of differentiation, cells were fixed with 4% paraformaldehyde and stained with a diazonium salt solution composed of fast violet blue salt and 4% naphthol AS-MX phosphate alkaline solution in an ALP kit, according to the manufacturer's protocol (Sigma-Aldrich Corp, St. Louis, MO, USA). Alkaline phosphatase enzymatic quantification was also performed as previously described [26], normalized to total protein content. After 28 days of differentiation, Alizarin Red and Von Kossa staining were performed to detect extracellular mineralization. The MSCs were fixed with 10% formalin for 30 min and stained with 1% silver nitrate solution under ultra- violet light for 45 min. The plates were then washed thoroughly with distilled water and treat with 3% sodium thiosulphate for 5 min. The presence of mineral deposits was indicated by the development of a black precipitate on the mineralized matrix. In the Alizarin red staining, the fixed cells were stained with a 0.2% Alizarin red S solution (Sigma-Aldrich Corp., St. Louis, MO, USA). The red staining represents calcium deposits on terminal differentiated cells.

### Adipogenic Differentiation and Chondrogenic Differentiation

MSCs were seeded in 12-well plate at a density of 10,000 cells/well for assessment of adipogenesis. MSCs were cultured in STEMPRO®Adipogenesis Differentiation Medium (Gibco, Invitrogen Corporation, USA). Oilred O staining was performed after 7 days of differentiation. For the chondrogenesis differentiation assay, generate micromass cultures by seeding 5-µl droplets of cell solution of 1.6×10^7^ cells/ml in the center of 12-well plate wells. After cultivating micromass cultures for 2 h under high humidity conditions, add warmed chondrogenesis media to culture vessels. After 14 days of cultivation, chondrogenic pellets can be processed for Alcian blue staining.

### Immunoprecipitation and western blot analysis

Cells were lysed in TNEN buffer containing 50 mM Tris-HCl (pH 7.5), 100 mM KCl, 1 mM EDTA, 0.5% NP-40, 0.1% Triton X-100, 1 mM Na_3_VO_4_, 10 mM NaF, 1 mM β-glycerol phosphate, 1 mM PMSF, and 1 µg/ml of aprotonin, leupeptin, and pepstatin. The immunoprecipitates or equal amounts of total cell lysates (50 µg) were resolved by SDS-PAGE and transferred to polyvinylidenedifluoride membranes (Millipore, Billerica, MA, USA). Proteins were detected by western blotting with the indicated antibodies. Antibodies against p-Smad1/5/8, Smad1, HA and His were purchased from Cell Signaling Technology, Inc.(Danvers, MA, USA). Antibodies against PDGFRα, Runx2, Atf4, Osx and β-actin were purchased from Santa Cruz Biotechnology, Inc. (Dallas, Texas, USA). Chloroquine and anti-Flag were purchased from Sigma. Anti- BMPRIA was from Abcam (Cambridge, MA USA). PDGF-AA, PDGF-AB, PDGF-BB and BMP-2 were obtained from PeproTech, Inc. (Suzhou, P.R.China). LDN-193189 was purchased from Selleckchem (Houston, TX, USA).

### Quantitation of western blot results

Western blot results were scanned with a molecular dynamics scanning densitometer. The relative levels of protein of interest were then determined by measuring the intensity of the corresponding bands. All values were averages of cell cultures at least 3 times and were normalized to the constitutive expression of Actin. In all the quantitation data, the values of empty vectors or untreated cells were set as 1.0.

### Quantitative RT-PCR and siRNA

Total RNA was extracted from cultured cells using Trizol reagent (Invitrogen, Carlsbad, CA,USA) according to manufacturer's protocol. cDNA was synthesized using a Quantscript RT Kit (Roche, Clare, Ireland). The detection and quantification of target mRNA was performed with real-time PCR using SYBR Green dye (Applied Biosystems, Foster City, USA), normalized to *GAPDH* expression levels. PCR primers used were depicted in Table S1. Small interference RNA (siRNA) targeting mouse PDGFRα obtained from Santa Cruz Biotechnology (sc-29444) were transfected into MSCs following the manufacturer's protocol. Down-regulation of PDGFRα was evaluated by western blot after a 48 h-transfection.

### Cell migration assays

The MSC migration assay was performed using transwell cell culture inserts (8-µm pore size; BD Bioscience, San Jose, CA,USA) for 24-well plates. 5×10^4^ cells in serum-free media were placed into the upper chamber and incubated at 37°C in 5% CO_2_ for 24 h, the cells remaining on the upper membrane were removed with cotton wool, whereas the cells that had migrated through the membrane were stained with 0.1% crystal violet. The number of migratory cells was counted in 5 random fields under a light scope (×200).

### Statistical analysis

Data are given as mean ± standard division (SD) of the results from three or four different samples in each item of the experiment. The cells from each sample were separately evaluated. Differences between two groups were measured by the Student's *t*-test. A *p* value less than 0.05 is defined as statistically significant difference.

## Results

### PDGF-AA activates Smad1/5/8 in MSC in a BMPRIA-dependent manner

**Figure 1:**
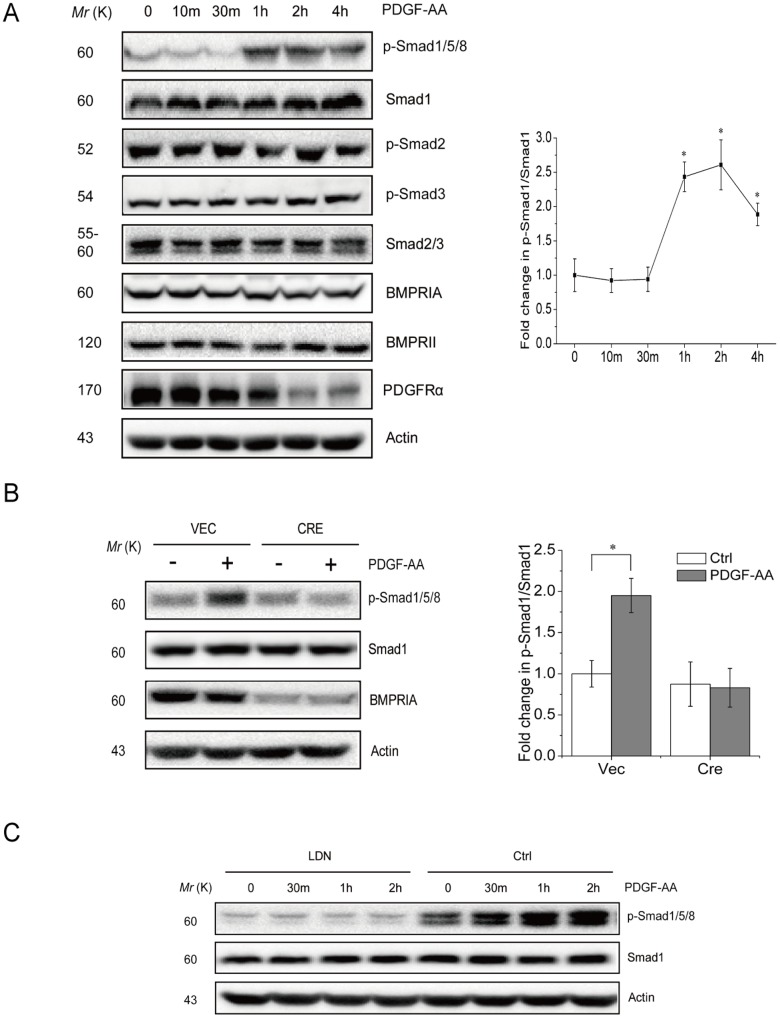
PDGF-AA activates BMP-Smad1/5/8 pathway via BMPRIA in MSC cells. A. PDGF-AA activates Smad1/5/8 but not Smad2/3 in MSC cultures. Primary MSC mouse cells were starved from serum for 4 h and then 25 ng/ml of PDGF-AA was added to the cultures. Cells were harvested at different time points and were lysed to analyze the activation of Smad1/5/8 and Smad2/3 by western blot. Right panel: quantitation data. B. BMPRIA deficiency diminished Smad1/5/8 activation at the basal level or in response to PDGF-AA. Primary mouse MSC cells were infected with control virus or virus expressing Cre and selected for 5 days, to knock out BMPRIA. These cells were serum starved and treated with PDGF-AA for 2 hrs. Cells were harvested to analyze the activation of Smad1/5/8 by western blot. Right panel: quantitation data. C. BMPRI inhibitor LDN-193189 diminished Smad1/5/8 activation at the basal level or in response to PDGF-AA. Primary mouse MSC cells were serum starved for 4 hrs, pretreated with LDN-193189 for 1 hr and then treated with PDGF-AA for different periods of time. Cells were harvested to analyze the activation of Smad1/5/8 by western blot. Data are means ±s.e.m. (n = 3 for all panels). * p<0.05, when compared to empty vectors or untreated cells.

Genetic studies have shown that PDGF molecules play important roles in MSC proliferation [27], [28], skeletal development and bone fracture healing, processes that involve bone morphogenetic proteins (BMPs) as well [23], [29]–[31]. We wanted to test whether there exists a connection between the PDGF and BMP signaling pathways in primary murine MSC cells. MSCs were isolated from the bone marrow of adult mice and they were able to adhere to the culture dish and proliferate for more than 5 generations. Moreover, they showed the capacity to differentiate into chondrocyte, adipocyte, or osteoblast (Fig. S1 and later results). We treated the primary mouse MSCs with PDGF-AA, AB, or BB for different periods of time and monitored Smad1/5/8 activation with specific antibodies. Since PDGF molecules are the major mitogens in the serum (up to tens of ng per ml) [32], [33], we starved MSCs from serum for 4 h before adding PDGF molecules. Under this condition, basal levels of Smad1/5/8 phosphorylation were detectable, which might be attributable to autocrine BMP molecules. Note that separation of the phosphorylated Smad1/5/8 SDS-PAGE gels was affected by the percentage of acrylamide and the running time. We found that PDGF-AA, but not AB or BB, significantly activated Smad1/5/8 (Fig. 1A and Fig. S2 and S3). However, PDGF molecules failed to activate Smad2/3, the TGFβ-responsive Smads (Fig. 1A), pointing to a specific link between PDGF-AA and BMP-Smad1/5/8 signaling. PDGF-AA could activate Smad1/5/8 in primary mouse embryonic fibroblasts as well (Fig. S4).

Smad1/5/8 phosphorylation is carried out by BMPRI [24]. We found that PDGF-AA-induced Smad1/5/8 activation requires BMPRIA as transient knockout of BMPRIA using retrovirus-expressed Cre in Bmpr1a^fl/fl^ MSC cells diminished PDGF-AA induced Samd1/5/8 activation (Fig. 1B). Moreover, inhibition of BMP receptor I with LDN-193189, a derivative of Dorsomorphin (compound C) and a highly selective BMPRI inhibitor [34], also diminished PDGF-AA-induced Smad1/5/8 activation (Fig. 1C). The results obtained from the studies on the BMPRI inhibitor and BMPRIA deficient cells suggest that PDGF-AA activates Smad1/5/8 signaling via BMPRIA in MSCs. However, we found that PDGF-AA did not alter the expression of BMPRIA and BMPRII at the protein levels (Fig. 1A), nor did it affect the protein levels of BMP2 in the culture medium, a major pro-osteogenesis agent (Fig. S5). These results suggest that PDGF-AA-induced activation of BMP-Smad1/5/8 signaling is not by altering the expression of BMPs or BMP receptors.

### PDGF-AA induces MSC osteogenic differentiation via Smad1/5/8

**Figure 2:**
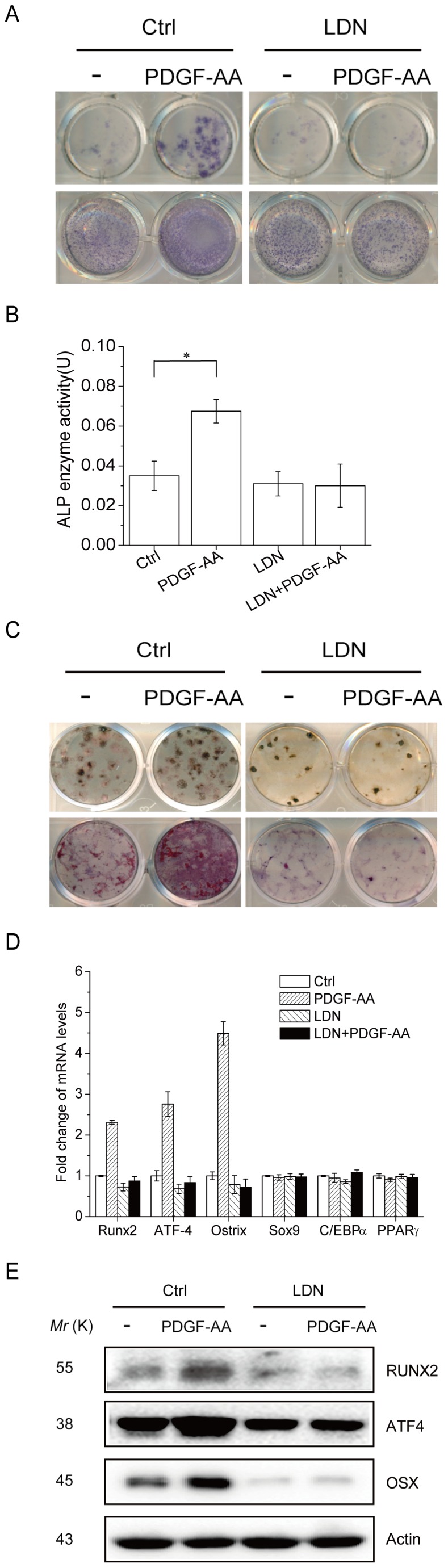
PDGF-AA promotes MSC osteogenic differentiation. A. PDGF-AA promotes ALP expression in MSC, which requires BMP-Smad1/5/8 signaling. Primary mouse MSC cells were cultured with or without PDGF-AA for 7 or 14 days. To test the effect of BMPRI activation, LDN-193189 was included in the culture medium. ALP was stained 7 or 14 days after culture. B. Quantitation data for ALP activities that were normalized to the total protein levels of each culture. C. PDGF-AA promotes bone mineralization in MSC, which requires BMP-Smad1/5/8 signaling. Primary mouse MSC cells were cultured in differentiation medium with or without PDGF-AA (changed every 3 days) for 28 days. To test the effect of BMPRI activation, LDN-193189 was included in the culture medium. Mineralization was stained with Von Kossa and Alizarin red method. D. PDGF-AA up-regulated the mRNA levels of Runx2, Atf4, and Osx via the BMP-Smad1/5/8 signaling, without affecting the mRNA levels of Sox9, C/EBPα, or PPARγ. Primary MSC cells were cultured with or without PDGF-AA for 7 days. To test the effect of BMPRI activation, LDN-193189 was included in the culture medium. Total RNA was isolated from the cells and realtime PCR was carried out to determine the mRNA levels of the six transcription factors. Data are means ±s.e.m. (n = 3 for all panels). * p<0.05, when compared to empty vectors or untreated cells. E. PDGF-AA up-regulated the protein levels of Runx2, Atf4, and Osx via the BMP-Smad1/5/8 signaling. The experiments were carried out as described in Fig. 2D and the protein levels of these proteins were determined by western blot.

To test the functions of PDGF-AA-induced Smad1/5/8 activation, we first looked at MSC osteogenic differentiation, a cellular event promoted by BMP-Smad1/5/8 signaling via transcription factors such as Runx2, Osterix, and Atf4 [25], [35]. We treated primary MSC with PDGF-AA for 7–28 days in differentiation medium and found that PDGF-AA induced ALP expression, evidenced by histological staining and quantitation assays (Fig. 2A and 2B), and promoted MSC mineralization, justified by Von Kossa or Alizarin red staining (Fig. 2C). The stimulatory activity of PDGF-AA on MSC osteogenic differentiation was diminished by co-treatment with BMPR inhibitor LDN-193189 (Fig. 2A–2C), suggesting that PDGF-AA promotes osteogenic differentiation via BMP-Smad1/5/8 signaling. In addition, we found that PDGF-AA up-regulates the mRNA levels of Runx2, Osterix, and Atf4, which were diminished by LDN-193189 treatment (Fig. 2D). Western blot analysis confirmed that PDGF-AA also increased the protein levels of Runx2, Osterix, and Atf4 in a BMP-Smad1/5/8-dependent manner (Fig. 2E). On the other hand, PDGF-AA did not affect the expression of Sox9, a master transcription factor required for MSC differentiation into chondrocytes, nor did PDGF-AA affect the expression of C/EBPα or PPARγ, two transcription factors required for MSC differentiation into adipocytes (Fig. 2D). These results suggest that PDGF-AA stimulates MSC differentiation into osteoblasts, via the BMP-Smad1/5/8-Runx2/Osx/Atf4 axis, without affecting its differentiation into chondrocytes or adipocytes. It is well known that PDGF-AA promotes MSC proliferation [8], [36]. We found that inhibition of BMPR with LDN-193189 showed no effect on this cellular event (Unpublished observations).

### PDGF-AA induces MSC migration via Smad1/5/8

**Figure 3:**
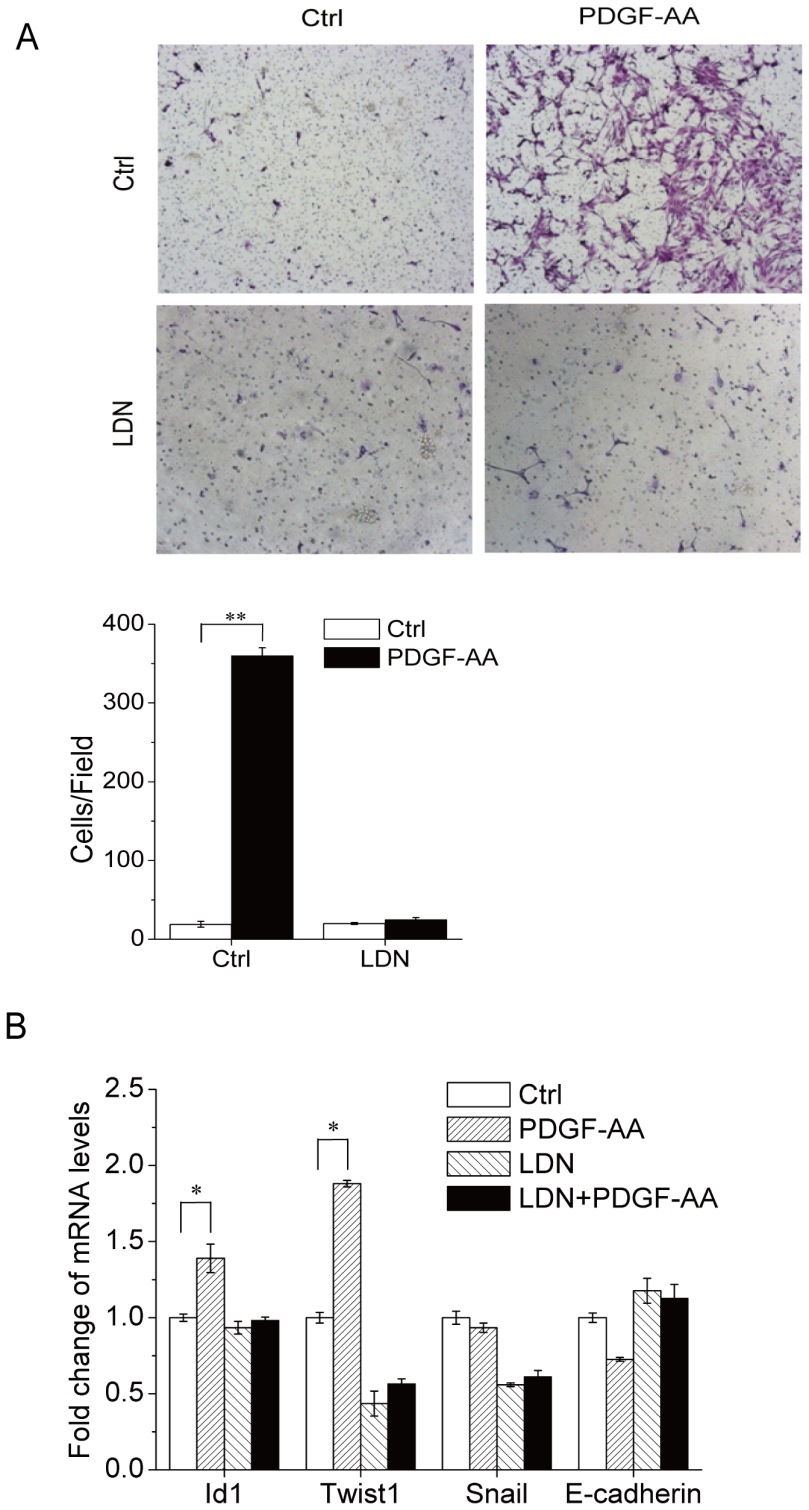
PDGF-AA promotes MSC migration via BMP-Smad1/5/8 signaling. A. Transwell experiments and quantitation data. Migration of MSCs was evaluated using a transwell chamber equipped with an 8-µm-pore filter membrane. Cells were plated at 5×10^4^ cells/well in serum-free media onto the upper compartment of the chamber and cultured in the absence or presence of PDGF-AA for 24 h. The filter membrane was removed, fixed with methanol, and stained with 0.1% crystal violet. Lower panel: the number of cells that had migrated to the lower surface of the filter membrane was counted in 5 random fields under a light scope (×200). B. PDGF-AA promotes expression of BMP-Smad1/5/8-controlled genes that regulate cell migration. Primary mouse MSC cells were cultured with or without PDGF-AA for 7 days. To test the effect of BMPRI activation, LDN-193189 was included in the culture medium. Total RNA was isolated from the cells and realtime-PCR was carried out to determine the mRNA levels of genes that are involved in regulating cell migration. Data are means±s.e.m. (n = 3). * p<0.05, **, P<0.01 when compared to empty vectors or untreated cells.

Both PDGF-AA and BMPs have chemotactic activities in various cell types [1]–[3]. Here we show that PDGF-AA could induce migration of MSC, justified by a transwell assay, which was inhibited by pretreatment with LDN-193189 (Fig. 3A), suggesting that part of the PDGF-AA's chemotactic activity on MSC cells can be mediated by BMP-Smad1/5/8 signaling. BMPs regulate cell migration via target genes including Id1, Twist1, Snail, and E-Cadherin [29], [37]–[39]. We found that the mRNA levels of BMP target gene Twist1 and Id1 were up-regulated by PDGF-AA in a BMP-Smad1/5/8-dependent manner (Fig. 3B), yet expression of other proteins involved in cell migration, e.g., Snail and E-Cadherin, was not altered. Moreover, it has been reported that BMPs could induce cell migration via Atf4 [40], which was also induced by PDGF-AA in a BMP-Smad1/5/8-dependent manner (Fig. 2D and 2E). These results suggest that the effect of PDGF-AA on MSC migration can be at least partially mediated by the BMP-Smad1/5/8 and its target genes including Id1, Twist1, and Atf4.

### PDGF-AA induced Smad1/5/8 activation requires lysosome-mediated degradation of PDGFRα

**Figure 4:**
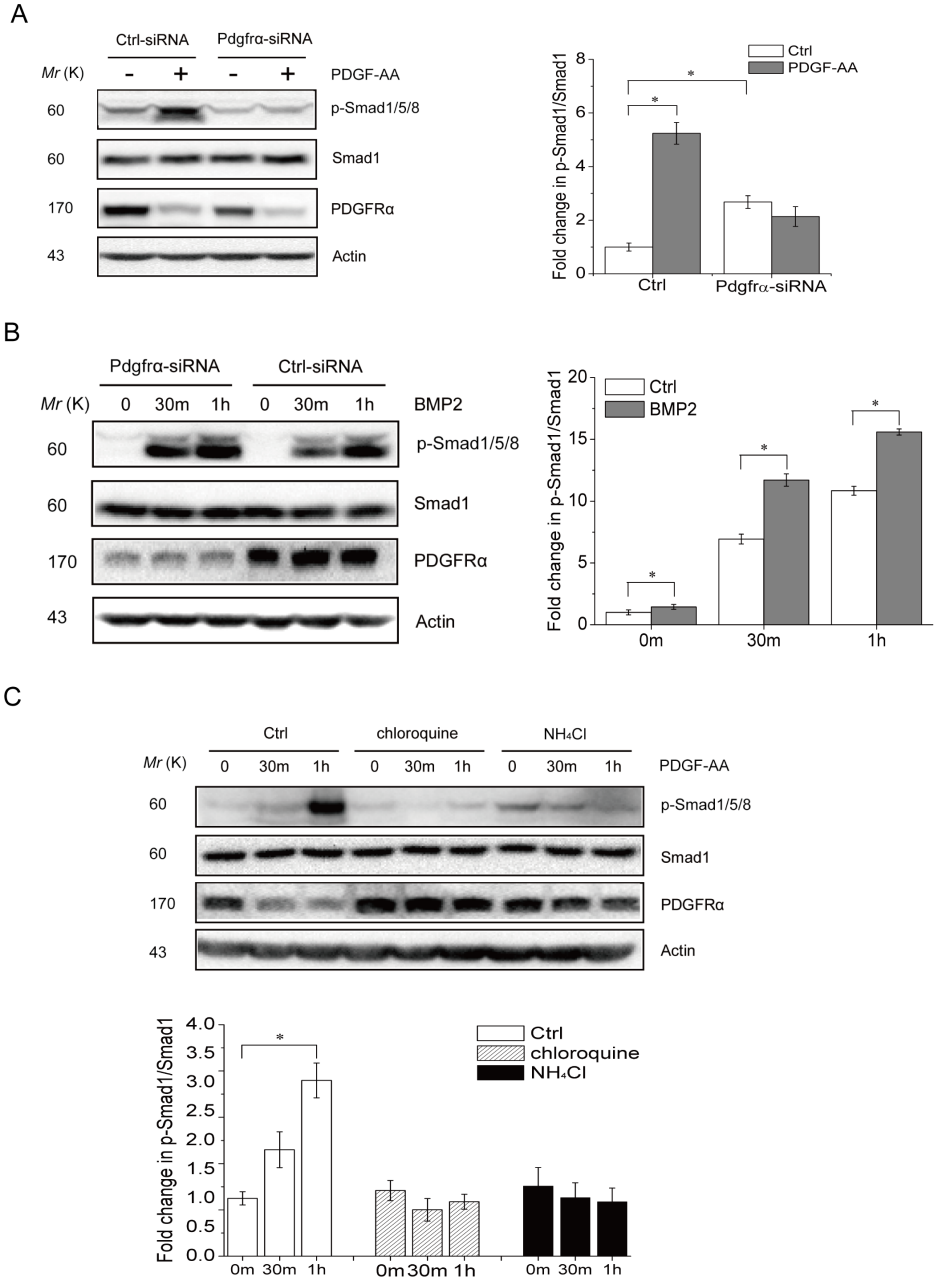
PDGFRα shows an inhibitory effect on Smad1/5/8 activation. A. PDGFRα knockdown enhanced Smad1/5/8 activation at the basal level but diminished PDGF-AA-induced Smad1/5/8 activation. Primary mouse MSC cells were transfected with control or siRNA against PDGFRα for 2 days. The cells were then treated with PDGF-AA for 2 hrs. Cells were harvested to analyze the activation of Smad1/5/8 by western blot. Right panel: quantitation data. B. PDGFRα knockdown enhanced Smad1/5/8 activation in response to BMP2. Primary mouse MSC cells were transfected with control or siRNA against PDGFRα for 2 days. The cells were then treated with 50 ng/ml of BMP2 for 0.5 or 1 hr. Cells were harvested to analyze the activation of Smad1/5/8 by western blot. Right panel: quantitation data. C. Inhibition of lysosome-mediated protein degradation diminished PDGF-AA-induced Smad1/5/8 activation. Primary mouse MSC cells were pretreated with chloroquine or NH_4_Cl for 6 h and then treated with PDGF-AA for 1 or 2 hrs. Cells were harvested to analyze the activation of Smad1/5/8 by western blot. Lower panel: quantitation data. Data are means ±s.e.m. (n = 3 for all panels). *, p<0.05, when compared to empty vectors or untreated cells.

Having shown the biological significance of PDGF-AA-induced Samd1/5/8 activation in MSCs, we wanted to understand the molecular mechanisms underlying Smad1/5/8 activation in response to PDGF-AA. We have excluded the possibility that Smad1/5/8 activation in response to PDGF-AA was caused by change in BMPs or BMPRs (Fig. 1A and Fig. S5). It is possible that PDGFR directly act on BMPRs and/or Smad1/5/8 to regulate their activation. PDGF-AA ligation to its receptors causes PDGFRα dimerization, which activates the downstream Erk and Akt1 pathways, followed by endocytosis and lysosome-mediated degradation of PDGFRα, which serve as a negative feedback mechanism [4], [5]. Down-regulation of PDGFRα was also observed in MSCs in response to PDGF-AA (Fig. 1A). To test whether down-regulation of PDGFRα plays a role in PDGF-AA-induced Smad1/5/8 activation, we knocked down PDGFRα with siRNA and found that this led to an increase in Smad1/5/8 activation at the basal level but a lack of further activation in response to PDGF-AA (Fig. 4A), suggesting that PDGFRα might inhibit BMP-Smad1/5/8 activation. Furthermore, we found that PDGFRα knockdown enhanced BMP2-induced Smad1/5/8 activation in MSCs as well (Fig. 4B), confirming that decreased levels of PDGFRα enhances BMP-Smad1/5/8 activation. If this is true, inhibition of PDGFR degradation should abolish PDGF-AA-induced Smad1/5/8 activation. PDGFRα, as well as many other receptor tyrosine kinases, is degraded in the lysosomes after activation, which could be inhibited by chloroquine or NH_4_Cl. We indeed found that inhibition of lysosome-mediated protein degradation by chloroquine or NH_4_Cl stabilized PDGFRα and diminished PDGF-AA-induced Smad1/5/8 activation (Fig. 4C). These results, taken together, indicate that PDGF-AA activates BMP-Smad1/5/8 signaling by down-regulating PDGFRα via lysosomes.

### PDGFRα interacts with BMPRIA

**Figure 5:**
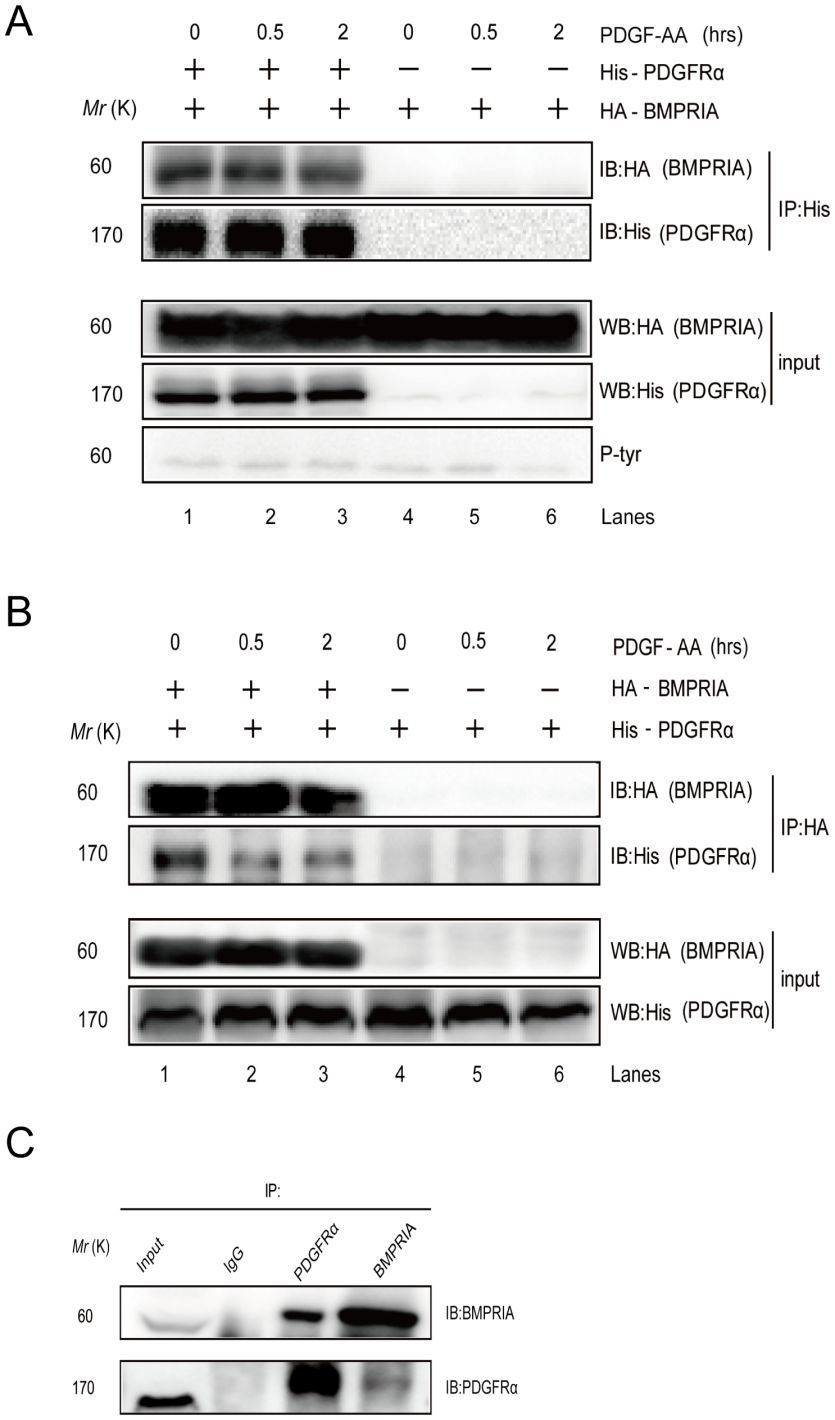
PDGFRα interacts with BMPRIA. A. PDGFRα (His tagged) was co-expressed with BMPRIA(HA tagged) in 293T cells. The cells were treated with 25 ng/ml PDGF-AA for 0.5 or 2 h or untreated. Cell lysates were divided into two parts for analysis of PDGFRα and BMPRIA expression by western blot, or immunoprecipitation of PDGFRα to test whether BMPRIA was brought down with PDGFRα. B. PDGFRα (His tagged) was co-expressed with BMPRIA(HA tagged) in 293T cells. The cells were treated with 25 ng/ml PDGF-AA for 0.5 or 2 h or untreated. Cell lysates were divided into two parts for analysis of PDGFRα and BMPRIA expression by western blot, or immunoprecipitation of BMPRIA to test whether PDGFRα was brought down with BMPRIA. C. Co-immunoprecipitation of endogenous PDGFRα and BMPRIA. Primary MSC cells were lyzed and cleaned lysates were incubated with IgG, anti-BMPRIA antibodies, or anti-PDGFRα antibodies overnight at 4°C. The immunoprecipitated proteins and their interacting proteins were detected by western blot.

How does decreased expression of PDGFRα enhance BMP-Smad1/5/8 signaling? PDGFRα may suppress BMPRIA-mediated Smad1/5/8 activation by interacting with and/or phosphorylating BMPRI, BMPRII, or Smad1/5/8. In co-expression experiments, we found that PDGFRα failed to tyrosine phosphorylate BMPRIA, BMPRII, or Smad1/5/8 (Fig. 5A and unpublished observations). In co-immunoprecipitation experiments, we found that ectopically expressed PDGFRα interacts with BMPRI as PDGFRα could bring down co-expressed BMPRIA at the basal levels (Fig. 5A and 5B). Moreover, BMPRIA could also bring down co-expressed PDGFRα at the basal levels (Fig. 5B). These results suggest that BMPRIA and PDGFRα constantly form a complex. On the contrary, PDGFRα did not bring down Smad1/5/8 or BMPRII (Unpublished observations). Further studies show that endogenous BMPRIA and PDGFRα also form a complex in co-immunoprecipitation assays (Fig. 5C).

### PDGFRα interferes with BMPRI-II interaction

**Figure 6:**
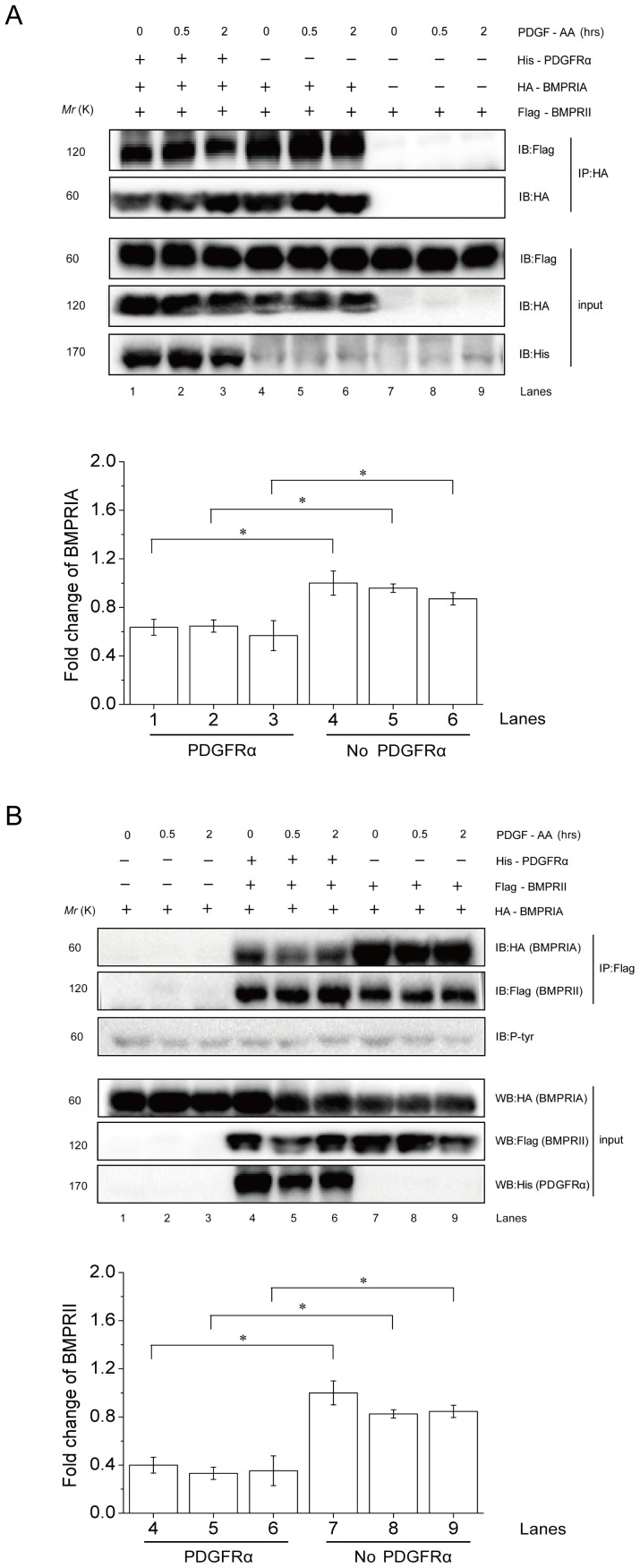
PDGFRα interferes with BMPRI-BMPRII interaction. A. PDGFRα (His tagged) was co-expressed with BMPRIA (HA tagged) and BMPRII (Flag tagged) in 293T cells. The cells were treated with 25 ng/ml PDGF-AA for 0.5 or 2 h or untreated. Cell lysates were divided into two parts for analysis of BMPRIA and BMPRII expression by western blot, or immunoprecipitation of BMPRIA to test whether the level of BMPRII brought down with BMPRIA was affected by expression of PDGFRα. Right panel: quantitation data. *, p<0.05, when compared to empty vectors transfected cells. B. PDGFRα (His tagged) was co-expressed with BMPRIA (HA tagged) and BMPRII (Flag tagged) in 293T cells. The cells were treated with 25 ng/ml PDGF-AA for 0.5 or 2 h or untreated. Cell lysates were divided into two parts for analysis of BMPRIA and BMPRII expression by western blot, or immunoprecipitation of BMPRII to test whether the level of BMPRIA brought down with BMPRII was affected by expression of PDGFRα. Right panel: quantitation data. *, p<0.05, when compared to empty vectors transfected cells.

The above results suggest that PDGFRα, by complexing with BMPRIA, may compete with BMPRII for BMPRIA binding and thus interfere with BMPRI-BMPRII interaction, which is required for BMPRI activation and Smad1/5/8 phosphorylation. To test this possibility, we carried out co-immunoprecipitation experiments and found that PDGFRα expression resulted in a decrease in BMPRI-II complex formation. In the presence of PDGFRα, BMPRIA brought down a reduced amount of BMPRII especially 30 min and 2 h after PDGF-AA treatment (Fig. 6A, compare lanes 4–6 to 1–3). Moreover, the presence of PDGFRα also decreased the levels of BMPRIA that was brought down by BMPRII (Fig. 6B, compare lanes 1–3 to 4–6). These results suggest that PDGFRα inhibits BMP-Smad1/5/8 activation by interacting with BMPRIA and interfering with BMPRI-BMPRII interaction.

## Discussion

PDGFs play important roles in bone development and bone fracture healing. PDGF-AA can be synthesized by bone cells and PDGF molecules are present at the bone fracture sites [6], [41]–[44]. The importance of PDGF-AA was also reflected by PDGFRα null mice, which showed defects in skeletal development and osteoblast survival [9]. Here we show that PDGF-AA is capable of promoting MSC migration and osteogenic differentiation, which might contribute to the physiological activities of PDGF-AA and PDGFRα in bone development and repair.

Our study shows that only PDGF-AA, but not BB or AB isoforms, has the ability to promote MSC differentiation and migration via activating BMP-Smad1/5/8 signaling. This is agreeable with the findings that PDGFRα null mice showed bone-related phenotypes whereas PDGFRβ null mice showed defects in hematopoiesis and blood vessel formation [1], [45], [46], and that PDGF-BB promotes osteoblast proliferation but represses osteogenic differentiation [17]. It was reported that PDGF-BB is synthesized and secreted by osteoclasts and inhibits osteogenic differentiation, suggesting that PDGF-BB might play a role in coupling bone resorption and bone formation [47]. All these results indicate that PDGF-AA and PDGF-BB might play distinct functions in MSC cells, which was attributable to their differential influence on BMP-Smad1/5/8 signaling. There are a few possibilities to explain why PDGF-AA but not PDGF-BB activates BMP-Smad1/5/8 signaling. Firstly, the difference between PDGF-AA and BB might be caused by the fact that PDGFRβ is usually recycled back to the cell surface [1]. However, we found that PDGF-BB could also result in down-regulation of PDGFRβ in MSC cells (Fig. S3), suggesting that this may not be the main reason. Secondly, previous studies showed that PDGFRβ is expressed at lower levels in osteoblast and PDGFRα is the major form [36], [48], [49]. It is thus possible that the change in PDGFRβ protein levels was not adequate to affect BMPRI-BMPRII interaction. Thirdly, it is possible that PDGFRβ might have a low affinity for BMPRI, which will not affect BMPRI-BMPRII interaction or BMP-Smad1/5/8 signaling. Future studies will be needed to distinguish these possibilities.

PDGFs and BMPs play critical roles in skeletal development, bone remodeling, and bone fracture repair [1], [50]. This study reveals a novel connection between PDGF-AA-PDGFRα and BMP-Smad1/5/8 signaling in MSC differentiation and migration. Previous studies have shown that growth factors including PDGFs inhibit BMP-Smad1/5/8 signaling by blocking Smad1/5/8 nuclear entry, which is mediated by Erks-mediated Smad1/5/8 phosphorylation at the linker region [51], [52], a region with multiple phosphorylation sites that integrate signaling from Wnt, Atm, and other pathways [53]–[57]. This study shows that PDGFRα also inhibits BMP-Smad1/5/8 signaling by interacting with BMPRI and interfering with BMPRI-BMPRII complex formation. During bone development and bone fracture healing, PDGF-AA-induced Erk1/2 activation promotes cell proliferation and its inhibition on Smad1/5/8 activation helps to block premature MSC differentiation and thus facilitates MSC expansion. Later on, Erk1/2 activation is attenuated, which relieves the inhibitory effect on BMP-Smad1/5/8 signaling. Furthermore, endocytosis-mediated PDGFRα turnover further stimulates BMP-Smad1/5/8 signaling by allowing BMPRI-BMPRII interaction, thus promoting osteogenic differentiation. Thus, PDGF-AA might initially augment MSC proliferation and later promote MSC osteogenic differentiation and migration, by modulating the BMP-Smad1/5/8 signaling. PDGF-AA released at the wound can help not only MSC recruitment and expansion but also osteogenic differentiation, and thus helping bone fracture healing via multiple ways.

Both BMPs and PDGF-AA play critical roles in embryonic development, adult tissue homeostasis, and tumorigenesis [2], [6], [41]–[44], [58]. Besides the bone, PGDF-AA and BMPs play important roles in intestinal villi and hair follicle formation, where they seem to have opposite functions. In adult, both PDGF-AA and BMPs are present in the stem cell niche for intestinal stem cells and hair follicle stem cells and they regulate the self-renewal and/or differentiation of these stem cells. In addition, Both PDGF-AA and BMPs are involved in tumorigenesis, e.g., glioma [1]. While PDGF-AA has pro-tumorigenesis activities, BMPs usually have anti-tumorigenesis activities. It will be interesting to test whether the interaction of PDGFRα and BMPI plays a role in these processes.

In summary, our studies reveal that PDGF-AA regulates MSC osteogenic differentiation and migration via BMP-Smad1/5/8 signaling. PDGF-AA exerts its functions by inducing feedback degradation of PDGFRα, which was shown to be a BMPRI-interacting protein that interferes with BMPRI and BMPRII interaction. The physical and functional interaction between BMPRIA and PDGFRα may play roles in other cellular and developmental processes that involve PDGF-AA and BMPs.

## Supporting Information

Figure S1 The mouse primary MSCs have the differentiation potential. MSCs were seeded in 12-well plate at a density of 10,000 cells/well for assessment of adipogenesis in the presence of adipogenesis differentiation medium. Oilred O staining was performed after 7 days of culture. For the chondrogenesis differentiation assay, we generated micromass cultures by seeding 5-µl droplets of cell solution of 1.6×10^7^ cells/ml in the center of 12-well plate wells. After 14 days of cultivation in the chondrogenesis medium, chondrogenic pellets were detected with Alcian blue staining.
(TIF)

Figure S2 PDGF-AB could not activate Smad1/5/8 or Smad2/3 in MSC cultures. Primary MSC cells were starved from serum for 4 hrs and then treated with 25 ng/ml PDGF-AB. Cells were harvested at different time points and lysed to analyze the activation of Smad1/5/8 and Smad2/3 by western blot. Three western blotting results and quantitation data from three repeated experiments were shown. Right panel: quantitation data.
(TIF)

Figure S3 PDGF-BB could not activate Smad1/5/8 or Smad2/3 in MSC cultures. Primary MSC cells were starved from serum for 4 hrs and then treated with 25 ng/ml PDGF-BB. Cells were harvested at different time points and lysed to analyze the activation of Smad1/5/8 and Smad2/3 by western blot. Three western blotting results and quantitation data from three repeated experiments were shown. Right panel: quantitation data.
(TIF)

Figure S4 PDGF-AA activates Smad1/5/8 in MEF cultures. Primary MEF cells were starved from serum for 4 hrs and then treated with 25 ng/ml PDGF-AA. Cells were harvested at different time points and lysed to analyze the activation of Smad1/5/8 by western blot.
(TIF)

Figure S5 PDGF-AA does not affect the protein levels of BMP2 in the culture media. To test whether PDGF-AA could induce the secretion of BMP2, we serum starved the cells for 4 hrs, and then added 25 ng/mL PDGF-AA to the culture medium of MSCs for 4 hrs. The culture medium was then collected to determine the concentration of BMP2 using a commercial kit (Cloud-Clone Corp) following the manufacturer's protocol.
(TIF)

Table S1 Oligonucleotide sequences for real-time PCR assays.
(DOCX)

Original Data S1 (RAR)
