# Supplementary figures and images for: PDGF-AA Promotes Osteogenic Differentiation and Migration of Mesenchymal Stem Cell by Down-Regulating PDGFRα and Derepressing BMP-Smad1/5/8 Signaling
Source: PLoS One. 2014 Dec 3;9(12):e113785. doi: 10.1371/journal.pone.0113785 (PMC4254917; doi:10.1371/journal.pone.0113785)

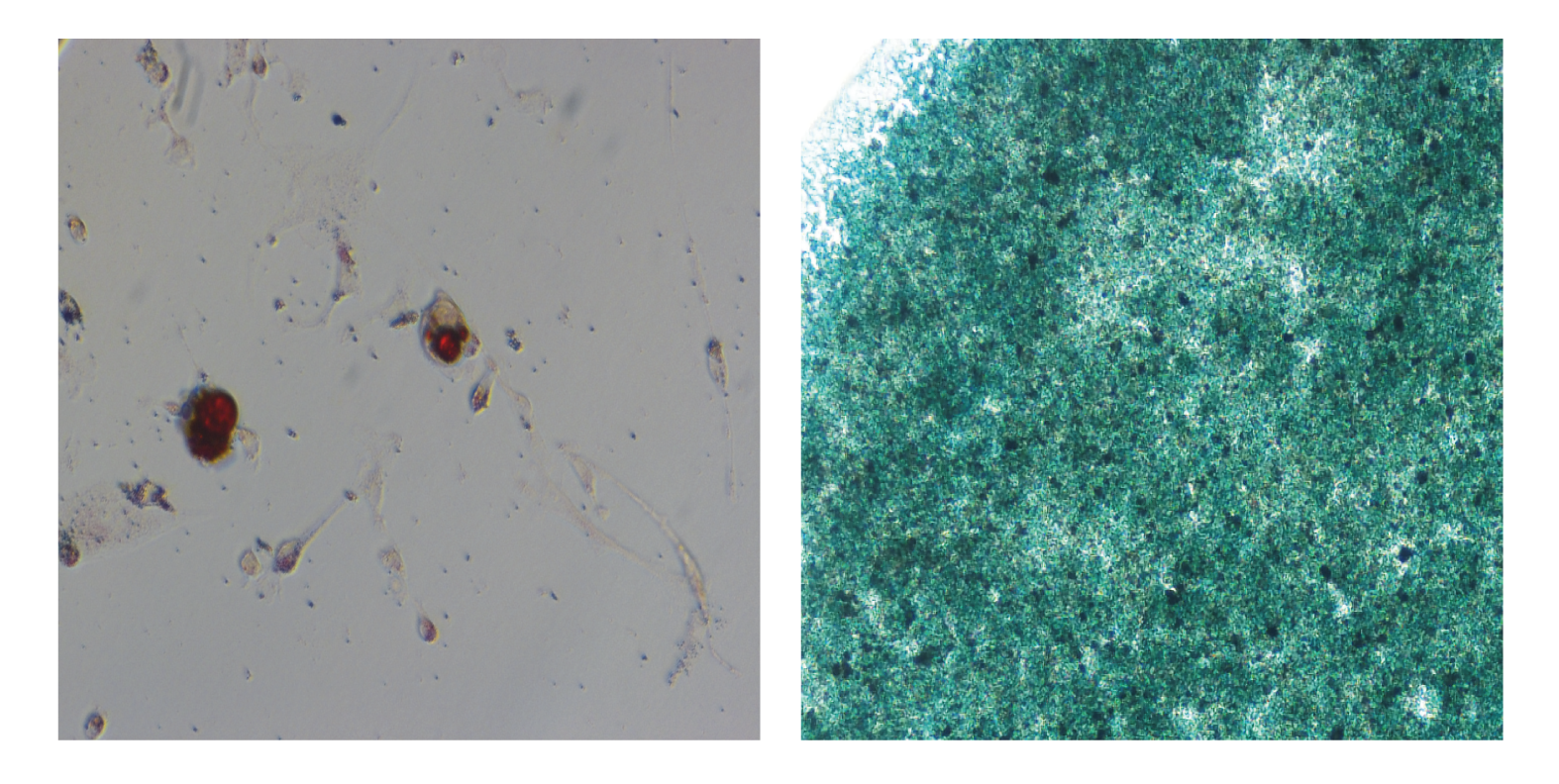

Supplement: Figure S1 — The mouse primary MSCs have the differentiation potential. MSCs were seeded in 12-well plate at a density of 10,000 cells/well for assessment of adipogenesis in the presence of adipogenesis differentiation medium. Oilred O staining was performed after 7 days of culture. For the chondrogenesis differentiation assay, we generated micromass cultures by seeding 5-µl droplets of cell solution of 1.6×10^7^ cells/ml in the center of 12-well plate wells. After 14 days of cultivation in the chondrogenesis medium, chondrogenic pellets were detected with Alcian blue staining. (TIF) [file pone.0113785.s001.tif]

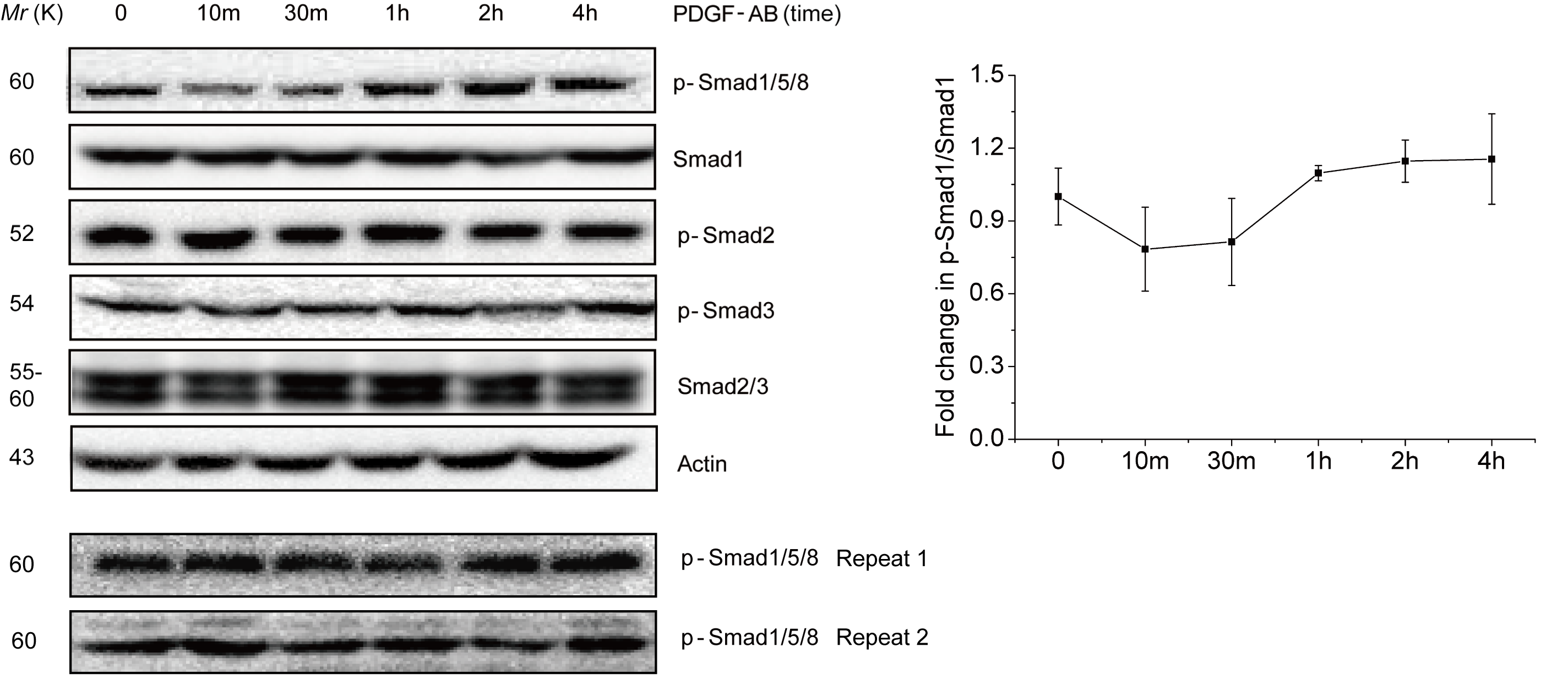

Supplement: Figure S2 — PDGF-AB could not activate Smad1/5/8 or Smad2/3 in MSC cultures. Primary MSC cells were starved from serum for 4 hrs and then treated with 25 ng/ml PDGF-AB. Cells were harvested at different time points and lysed to analyze the activation of Smad1/5/8 and Smad2/3 by western blot. Three western blotting results and quantitation data from three repeated experiments were shown. Right panel: quantitation data. (TIF) [file pone.0113785.s002.tif]

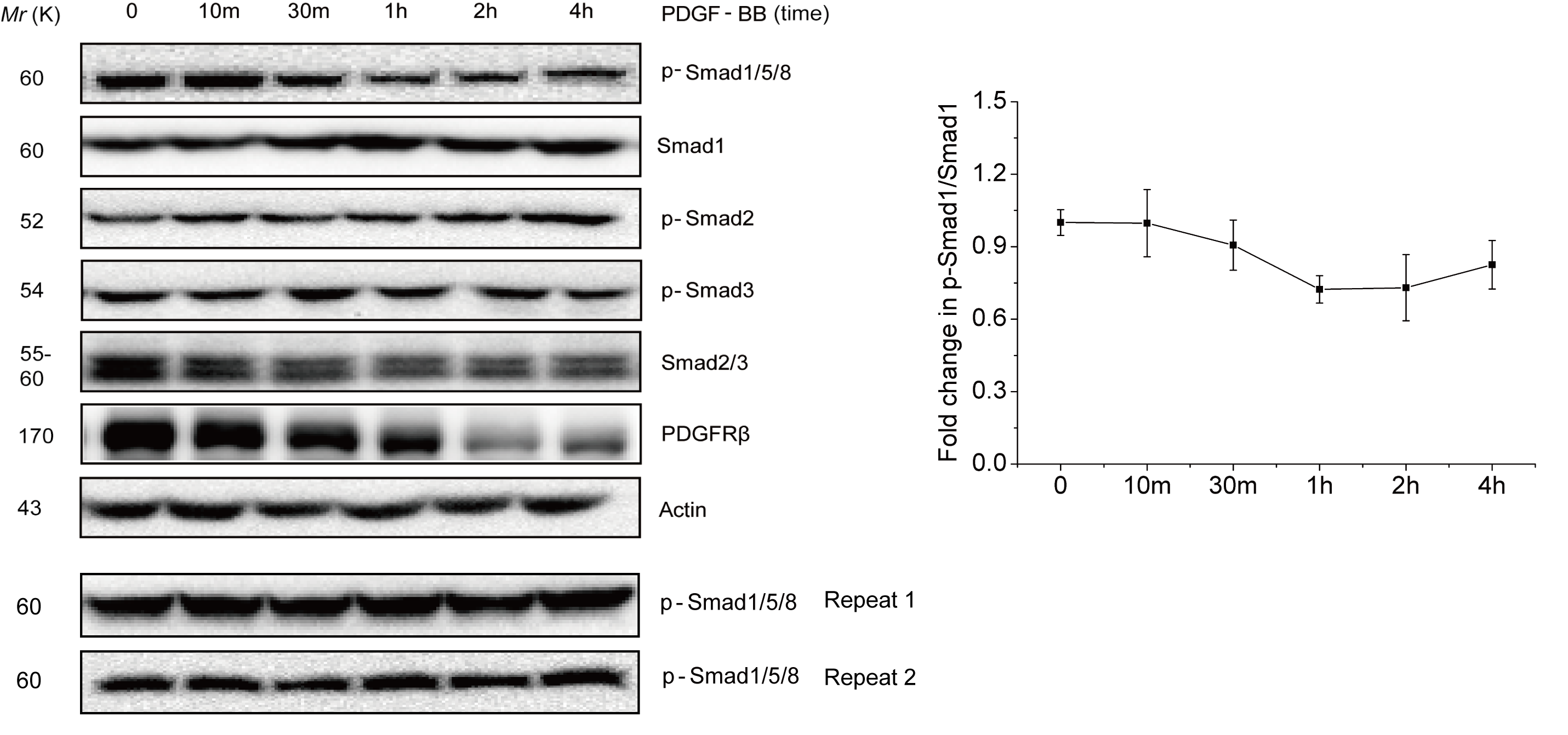

Supplement: Figure S3 — PDGF-BB could not activate Smad1/5/8 or Smad2/3 in MSC cultures. Primary MSC cells were starved from serum for 4 hrs and then treated with 25 ng/ml PDGF-BB. Cells were harvested at different time points and lysed to analyze the activation of Smad1/5/8 and Smad2/3 by western blot. Three western blotting results and quantitation data from three repeated experiments were shown. Right panel: quantitation data. (TIF) [file pone.0113785.s003.tif]

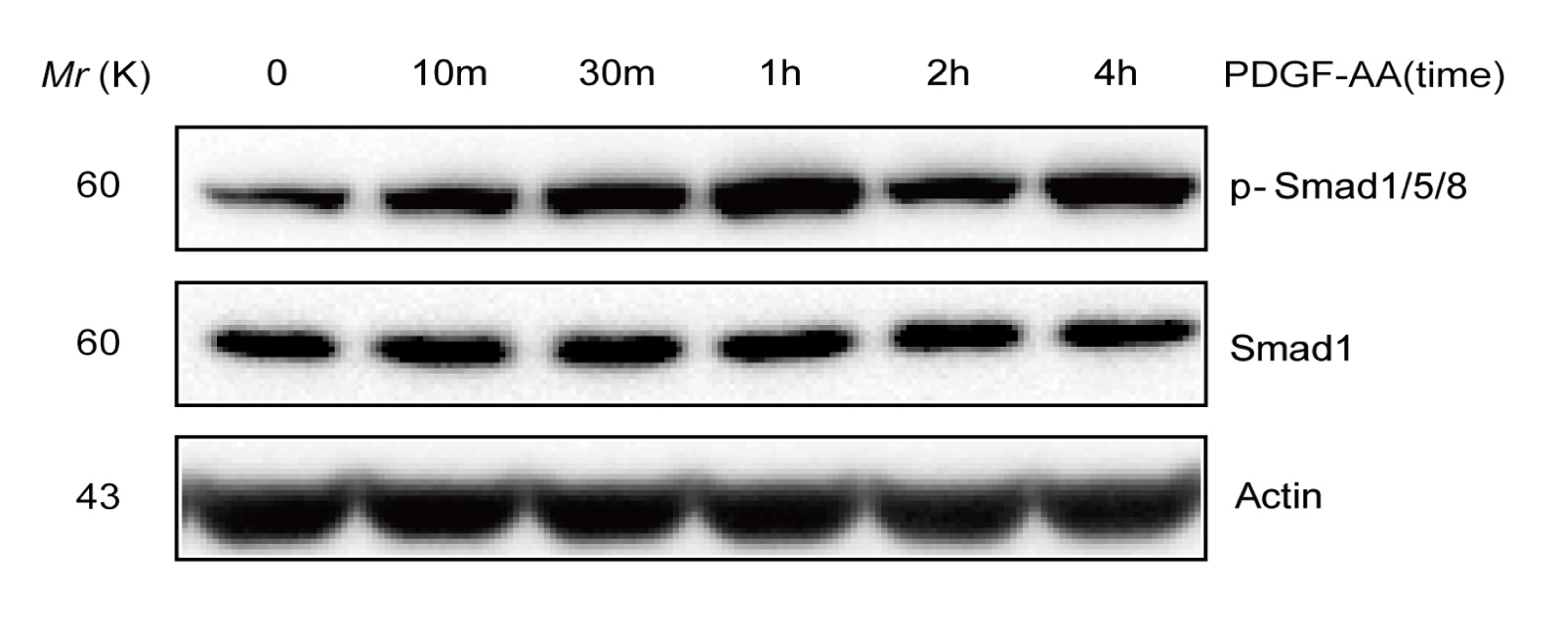

Supplement: Figure S4 — PDGF-AA activates Smad1/5/8 in MEF cultures. Primary MEF cells were starved from serum for 4 hrs and then treated with 25 ng/ml PDGF-AA. Cells were harvested at different time points and lysed to analyze the activation of Smad1/5/8 by western blot. (TIF) [file pone.0113785.s004.tif]

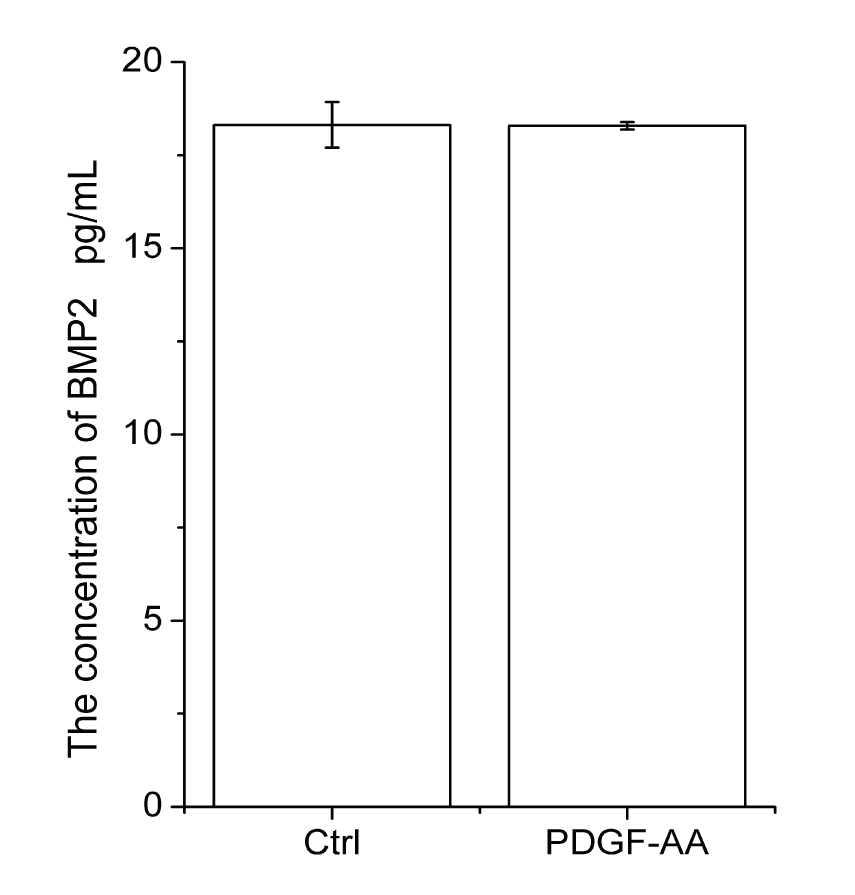

Supplement: Figure S5 — PDGF-AA does not affect the protein levels of BMP2 in the culture media. To test whether PDGF-AA could induce the secretion of BMP2, we serum starved the cells for 4 hrs, and then added 25 ng/mL PDGF-AA to the culture medium of MSCs for 4 hrs. The culture medium was then collected to determine the concentration of BMP2 using a commercial kit (Cloud-Clone Corp) following the manufacturer's protocol. (TIF) [file pone.0113785.s005.tif]
