# Supplementary material for: PDGF-AA Promotes Osteogenic Differentiation and Migration of Mesenchymal Stem Cell by Down-Regulating PDGFRα and Derepressing BMP-Smad1/5/8 Signaling
Source: PLoS One. 2014 Dec 3;9(12):e113785. doi: 10.1371/journal.pone.0113785 (PMC4254917; doi:10.1371/journal.pone.0113785)
Supplement: Table S1 — Oligonucleotide sequences for real-time PCR assays. (DOCX) [file pone.0113785.s006.docx]

**Supplementary Tables**

**Table S 1. Oligonucleotide sequences for real-time PCR assays**

| **Genes** | **Forward** | **Reverse** |
| --- | --- | --- |
| **Runx2** | 5’ tttagggcgcattcctcatc 3’ | 5’ tgtccttgtggattaaaaggacttg 3’ |
| **ATF-4** | 5’ CTCGGCCCAAACCTTATG 3’ | 5’ CTTCTATCAGGTCTTTCAGATACT 3’ |
| **Osterix** | 5’ actcatccctatggctcgtg 3’ | 5’ ggtagggagctgggttaagg 3’ |
| **Sox9**  **C/EBPα**  **PPARγ**  **Id1** | 5’ AGTCCCAGCGAACGCACATCA 3’  5’ TGGACAAGAACAGCAACGAG 3’  5’ ACTGCCTATGAGCTCTTCAC 3’  5’ GCGAGATCAGTGCCTTGG 3’ | 5’ GTCGTATTGCGAGCGGGTGAT 3’  5’ AATCTCCTAGTCCTGGCTTG 3’  5’ CAATCGGATGGTTCTTCGGA 3’  5’ CTCCTGAAGGGCTGGAGTC 3’ |
| **Twist1** | 5’ TGGACAGAGATTCCCAGAGG 3’ | 5’ TTCCTGTCAGTGGCTGATTG 3’ |
| **Snail** | 5’ GAGGACAGTGGCAAAAGCTC 3’ | 5’ TCGGATGTGCATCTTCAGAG 3’ |
| **E-cadherin** | 5’ CAAGGACAGCCTTCTTTTCG 3’ | 5’ TGGACTTCAGCGTCACTTTG3’ |
| **β-actin** | 5’ CTAAGGCCAACCGTGAAAAG 3’ | 5’ ACCAGAGGCATACAGGGACA 3’ |
